# Environmental Effects on Bacterial Community Assembly in Arid and Semi-Arid Grasslands

**DOI:** 10.3390/microorganisms13081934

**Published:** 2025-08-19

**Authors:** Shenggang Chen, Yaqi Zhang, Jun Ma, Mingyue Bai, Yinglong Chen, Jianbin Guo, Lin Chen

**Affiliations:** 1 School of Soil and Water Conservation, Beijing Forestry University, Beijing 100083, China; shenggangchen@bjfu.edu.cn (S.C.);; 2 Key Laboratory for Restoration and Reconstruction of Degraded Ecosystem in Northwest China of Ministry of Education, School of Ecology and Environment, Ningxia University, Yinchuan 750021, China; 3 School of Forestry and Prataculture, Ningxia University, Yinchuan 750021, China; 4 School of Agriculture and Environment, The University of Western Australia, Perth, WA 6009, Australia

**Keywords:** arid and semi-arid grasslands, soil depth, bacterial diversity, community assembly, deterministic vs. stochastic processes

## Abstract

Studying the effects of environmental factors on microbial community assemblies is crucial for understanding microbial biodiversity and ecosystem processes. Although numerous studies have explored the spatial patterns of microbial communities in surface soils, bacterial community distributions in subsurface layers remain poorly understood. We investigated multiple community metrics of soil bacteria in arid and semi-arid grasslands in China, and the V4 region of 16S rDNA was analyzed using soil property measurements, fluorescent PCR, and high-throughput sequencing techniques. Specifically, copiotrophic taxa dominate the topsoil, whereas oligotrophic taxa are prevalent in nutrient-limited subsoil. Bacterial diversity decreases from the topsoil to subsoil, and bacterial distribution and ecological community composition exhibit a strong dependence on environmental factors. Moreover, microbial interaction networks demonstrated a progressive simplification with increasing soil depth: topsoil communities displayed higher modularity and a greater prevalence of positive interactions, whereas subsoil networks were significantly less complex. Null model analyses evidenced assembly mechanisms: deterministic processes (particularly homogeneous selection) dominated the bacterial community assembly, but their influence weakened with depth, whereas stochastic processes (e.g., dispersal limitation) increased progressively from the topsoil to subsoil. The PLS-PM analysis demonstrated that the relative influence of abiotic factors (e.g., climatic conditions and nutrient availability), biotic factors (interspecific interactions), along with drift and dispersal limitations on fungal community assembly exhibited depth-dependent patterns. This study provides novel insights into the vertical stratification of bacterial community in arid and semi-arid grasslands, and advances our understanding of pedogenic process under climate change and microbial adaptive strategies in heterogeneous soil environments.

## 1. Introduction

Soil harbors a highly diverse microbiota that plays fundamental roles in sustaining soil ecosystems [1]. These microbial communities are key drivers of biogeochemical processes, regulating nutrient cycling and ecosystem functioning on a global scale [2]. While horizontal variations in soil microbial composition have been extensively studied—particularly in Eastern China’s ecosystems [3]—far less is known about microbial stratification along vertical soil profiles, where distinct ecological patterns emerge [4]. The assembly of soil microbial communities is shaped by a complex interplay of biotic and abiotic factors. For instance, bacterial diversity has been shown to strongly influence community structure [5], whereas environmental variables such as pH, organic matter, and nutrient availability are critical determinants of bacterial composition [6,7,8]. These microbial communities, in turn, govern essential soil processes, including mineralization, organic matter decomposition, and nutrient transformation, with profound implications for plant productivity and ecosystem health [9]. Yet, microbial dynamics exhibit pronounced heterogeneity across soil depths [10]. Vertical stratification introduces additional complexity, as deeper soil layers exhibit starkly different conditions—reduced carbon availability, nutrient limitation, and the dominance of oligotrophic taxa adapted to these constraints [11,12,13]. Additionally, ecological processes structuring microbial communities vary significantly with depth, being influenced by factors such as parent material, soil type, and land-use practices [14,15]. Emerging evidence suggests that deeper soil layers (>20 cm) harbor functionally distinct microbial assemblages that may contribute disproportionately to ecosystem multifunctionality [16,17]. The influence of soil depth on bacterial community composition is currently unclear, as are the main influencing factors.

Understanding the co-occurrence patterns of bacterial communities represents a pivotal approach in soil biogeography [18,19,20]. By analyzing community network characteristics—including degree, modularity, and clustering coefficient—we can decipher niche overlap among species and identify keystone taxa that drive ecosystem functioning [21]. According to the assessment of microbial presence–absence or abundance among taxa, this co-occurrence approach emphasizes the biological relationships between positive and negative, as known as co-occurrence and co-exclusion [22]. Network analyses of co-occurrence have become a powerful tool for unravelling the processes involved in the assembly of microbes in soils [23], with recent studies demonstrating that environmentally specific co-occurrence patterns are a defining feature of soil microbiomes [24]. For instance, in Swiss wheat fields, keystone taxa within co-occurrence networks have been strongly linked to soil phosphorus content, bulk density, and pH [19], underscoring the ecological relevance of these interactions.

A key discussion in microbial ecology focuses on the comparative roles of deterministic (niche-driven) and stochastic processes in governing bacterial community assembly [25]. In addition to environmental filtering, microbial interactions also contribute to the assembly process [26,27]. Emerging evidence reveals striking contrast across ecosystems: in subtropical Huangshan forests, the bacterial assembly was predominantly stochastic across soil layers [25], whereas in Mount Gongga’s forests, deterministic processes prevailed, regardless of altitude or season [28,29]. Yet, paddy soils exhibited stochastic dominance throughout 0–40 cm profiles [30]. Similarly, grassland studies have found that deterministic selection strengthens with soil depth [31], yet comprehensive studies across arid and semi-arid grasslands are conspicuously absent from the literature; critical knowledge gaps persist—particularly in heterogeneous environments like arid and semi-arid grassland ecosystems, where microbial assembly mechanisms remain poorly resolved [32]. Moreover, research on bacterial community distribution in grassland subsurface soils is strikingly limited [33,34], leaving several crucial blind spots in our understanding of arid and semi-arid grasslands: (1) how the strength of microbial community interactions varies with soil depth, (2) how these changes affect bacterial community assembly, and (3) how environmental factors influence biological interactions and indirectly affect bacterial community assembly. These unknowns are particularly prominent in understanding bacterial community assemblies in arid and semi-arid grasslands.

Ningxia is located in the arid and semi-arid transition zone and is one of the three pilot provinces of the ‘Research on Climate Change Adaptation in China’ project. The grasslands in this region extend from north to south, resulting in a large mean annual precipitation (MAP) gradient and varied grassland types. This eco-climatic gradient provides a natural laboratory to study microbial distributions on a large scale. By utilizing high-throughput 16S rRNA sequencing and detailed soil physicochemical analyses, we examined spatial and environmental variations in bacterial communities across 66 soil profiles, covering three soil layers, 0–20 cm (topsoil), 20–40 cm (middle layer), and 40–100 cm (subsoil). We formulate the following hypothesis: (1) the bacterial community exhibits distinct composition, diversity, and co-occurrence network patterns among different soil horizons; (2) the bacterial coexistence network in the topsoil is more complex and the intensity of interactions between species is greater than those in the subsoil; and (3) soil environmental factors are crucial factors influencing the assembly process of bacterial communities. This work provides the first comprehensive examination of vertical microbial stratification in arid and semi-arid grasslands, offering novel insights into depth-dependent assembly mechanisms. Our findings not only advance the fundamental understanding of soil microbial ecology, but also provide critical reference value for predicting ecosystem responses to environmental change and informing conservation strategies in arid and semi-arid regions.

## 2. Materials and Methods

### 2.1. Study Area

The study area, located in the Ningxia Hui Autonomous Region of China, spans 66,400 km^2^ and lies between the Loess Plateau and the Mongolian Plateau. It is defined by the coordinates 35°14′–39°23′ N, 104°17′–107°39′ E [35]. Encompassing nearly all major grassland types found in northern China: alpine meadow (AM), typical grassland (TG), desert grassland (DG), and grasslandization desert (GD). In the southern Loess Plateau, AM and TG are dominant, with TG primarily distributed near Guyuan City (e.g., Yunwu Mountain Grassland Nature Reserve). This region experiences a semi-arid climate, with annual precipitation ranging from 300 to 400 mm, where drought-resistant perennial tufted grasses predominate. In contrast, AM primarily occurs on the shady slopes and in the valleys of the Liupan Mountains and other mountainous areas, where moisture availability is higher and the climate is relatively humid, with annual precipitation ranging from 400 to 600 mm. This vegetation type consists mainly of perennial and rhizomatous grasses, which thrive in moderately arid conditions. DG is distributed across the central and northern parts of Ningxia, which is the overland of grassland and desert, with an arid climate; annual precipitation is generally around 200–300 mm, and vegetation cover is 40–60%, with dry perennial grasses dominating and small dry shrubs participating. Meanwhile, GD occupies the northern and northwestern parts of Ningxia, adjacent to the Tengger Desert and Mao Wusu Desert; the climate is extremely arid, the annual precipitation is usually less than 200 mm, vegetation is sparse (<30%), and super-arid shrubs and small half-shrubs are dominant [36,37]. This diversity offers a unique natural laboratory to investigate how varying ecosystems respond to climatic shifts.

### 2.2. Site Selection and Soil Sampling

**Figure 1:**
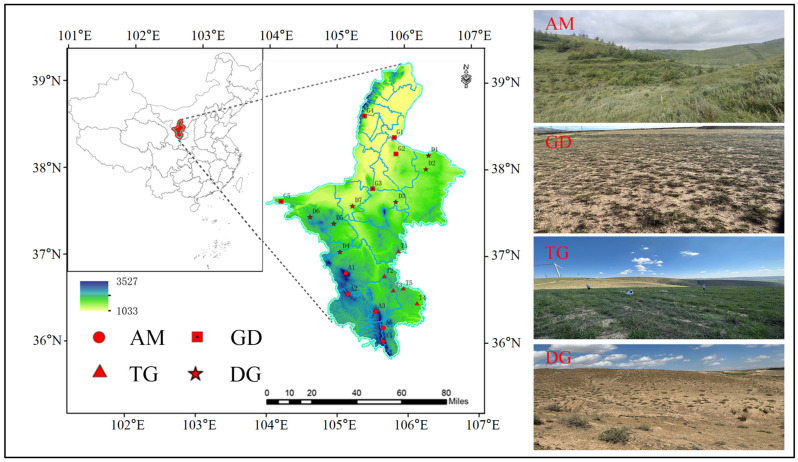
Distribution of the sampling sites in different grassland types. AM: alpine meadow, GD: grasslandization desert, TG: typical grassland, DG: desert grassland.

To capture the ecological diversity along the precipitation gradient from south to north, four grassland types were selected (Figure 1). The number of sampling sites for each grassland type was proportional to their respective areas: MS (5 sites), DS (7 sites), TS (5 sites), and SD (5 sites). The latitude, longitude, and elevation of each site were recorded, and mean annual temperature (MAT) and annual precipitation (MAP) were obtained from the databases (http://www.worldclim.org/). At each site, three replicate plots (20 m × 20 m) were randomly established. Within each plot, soil samples were collected from three depth intervals (0–20 cm, 20–40 cm, and 40–100 cm) using a five-point sampling method, with five cores extracted per depth. The cores from each depth were homogenized to form a composite sample. Each composite sample was then divided into three subsamples: one preserved at –80 °C for DNA extraction, one stored at 4 °C for physicochemical analysis, and the third reserved for microbial measurements. Additionally, vegetation surveys were conducted in three randomly selected 1 × 1 m subplots within each plot (Table S1).

### 2.3. Soil Physicochemical Properties

The core method was utilized to determine soil bulk density (BD) with a 100 cm^3^ ring knife (height: 5 cm; diameter: 5.05 cm) [38]. To quantify soil water content (SWC), fresh soil samples were oven-dried at 102 °C until their weight remained constant [39]. A pHS-3C pH meter was utilized to measure soil pH at a soil:water ratio of 1:2.5 (*w*/*v*) [40]. SOC was quantified using the K_2_Cr_2_O_7_ external heating method, followed by titration with 0.1 M FeSO_4_ [41]. The concentration of total nitrogen (TN) was measured employing the Kjeldahl method with a Kjeltec 8400 analyzer (FOSS, Copenhagen, Denmark). Available nitrogen (AN) was determined using alkaline hydrolysis diffusion. The concentration of total phosphorus (TP) was determined using an ultraviolet spectrophotometer (Shimadzu, Tokyo, Japan) following wet digestion with a mixture of H_2_SO_4_ and HClO_4_. Available phosphorus (AP) was determined using sodium bicarbonate extraction. Available potassium (AK) was extracted using a 1 mol/L ammonium acetate solution (pH 7.0) and subsequently quantified by atomic absorption and emission spectrophotometry. Total carbon (TC) was analyzed using the potassium dichromate external heating method [42,43]. A conductivity meter was employed to measure the soil electrical conductivity (EC).

### 2.4. Illumina Sequencing Analysis of 16S rRNA Gene Amplicons

The genomic DNA was extracted from 0.5 g soil samples using a MoBio PowerSoil DNA isolation kit (MoBio Laboratories, Inc., Carlsbad, CA, USA). The DNA concentration was measured using a NanoDrop 2000 UV-vis spectrophotometer (Thermo Fisher, Shanghai, China), and quality was assessed by gel electrophoresis. The V3–V4 region [44] of bacterial and fungal genes was amplified in triplicate using the primer pair 338F (5′-ACTCCTACGGGAGGCAGCA-3′) and 806R (5′-GGACTACHVGGGTWTCTAAT-3′). Following purification, PCR products from all samples were normalized to equimolar concentrations prior to sequencing. The Illumina NovaSeq PE250 platform (Illumina, CA, USA) was employed for high-throughput sequencing. The sequences were analyzed with the amplicon sequence variants (ASVs) approach in the QIIME2 pipeline (ver. 2018.2). We denoised the data and joined paired reads using the Divisive Amplicon Denoising Algorithm 2 method (DADA2) [45]. 16S sequences were aligned using mafft, and trees were built using Fast Tree (http://www.microbesonline.org/fasttree/; accessed on 5 July 2022) within QIIME2. After removing singletons, 17,187 ASVs were obtained for all soil samples and all samples were rarefied as the minimum number of sequences (16,611) for downstream analysis. The bacterial DNA content in the soil samples was quantified through qPCR amplification of 16S rRNA genes. The reactions were performed in triplicate on a 7500 real-time PCR system (Thermo Fisher, Shanghai, China), following the methodology outlined in previous studies [46].

### 2.5. Cooccurrence Network Analysis

This study employed network analysis to construct co-occurrence networks at the genus taxonomic level. Specifically, we first applied stringent selection criteria, focusing only on phyla with mean relative abundances exceeding 1% and subsequently selecting the top 200 most abundant genera within these phyla. This selective strategy ensured that our networks captured ecologically relevant taxa while minimizing groups from low-abundance organisms. Using the well-established WGCNA package [47] in R (version 4.1.2), we generated highly reliable Spearman correlation matrices and derived co-occurrence networks for each soil horizon, implementing strict statistical thresholds (*p* < 0.05 and correlation coefficient > 0.8) to guarantee robust associations. The resulting networks were meticulously visualized using Gephi (ver.0.10), enabling clear interpretation of microbial interaction patterns (http://gephi.github.io/; accessed on 5 July 2022). Network topology properties, calculated using the igraph package (http://igraph.org), offered quantitative measures of microbial community organization and stability. Furthermore, by extending this sophisticated network approach to taxon–environment relationships, we established a framework for linking microbial community structure to environmental factors.

### 2.6. Estimation of Community Assembly Processes

Following previous research, we employed Stegen’s null model frameworks to estimate the processes governing community assembly [48]. We quantified the relative importance of distinct ecological processes in soil bacterial community assembly by combining the β-nearest taxon index (β-NTI) and the Bray–Curtis-based Raup–Crick metric (RC-bray). The processes included homogeneous selection (β-NTI < −2), variable selection (β-NTI > 2), homogeneous dispersal (RC-bray < −0.95 and |β-NTI| < 2), dispersal limitation (RC-bray > 0.95 and |β-NTI| < 2), and drift (|RC-bray| < 0.95 and |β-NTI| < 2).

### 2.7. Statistical Analysis

All statistical and visualization workflows were implemented in R (v4.1.2), ensuring reproducibility and transparency. Microbial alpha diversity—quantified via Shannon diversity and phylogenetic diversity metrics (vegan package)—was systematically compared across layers using one-way ANOVA followed by Duncan’s post hoc tests (*p* < 0.05), providing robust statistical validation of depth-dependent trends.

To uncover community turnover dynamics, this article used non-metric multidimensional scaling (NMDS), based on Bray–Curtis (taxonomic) and weighted UniFrac (phylogenetic) distances. The phyloseq and vegan packages were used for advanced ecological inference [49]. The significance of horizon-driven beta diversity differences was further confirmed through ANOSIM (analysis of similarities) and ADONIS (analysis of dissimilarities).

The slopes of the distance–decay relationships of the microbial community were calculated at three soil depths: topsoil, middle layer, and subsoil. The slope coefficient at each depth was calculated based on the equation below:

(1) $$Ln\left(C\right)=\alpha +\beta \times Ln(E/G)$$

where C is the community dissimilarity, E/G is the environmental dissimilarity or geographical distance, α is an intercept value and β is the slope coefficient of the distance-decay curve [50]. The geographic distance was calculated with the R (ver. 3.6.2) package ‘geosphere’ [51].

In the PLS-PM analysis, all variables were standardized using Z-score transformation (Origin, version 2021) to approximate a normal distribution and equalize variances, facilitating comparison, testing, and accurate modeling for information interpretation. We used PLS-PM to evaluate the effects of environmental factors, spatial distance and taxa associations, as well as their indirect effects, on betaNTI. Piecewise structural equation modeling (SEM) was performed using the ‘piecewiseSEM’ package in R (ver. 3.6.2).

To investigate the relative importance of biotic associations and habitat selection in determining the system of determining phylogenetic turnover in each soil layer, a multiple regression analysis based on the distance matrix was performed to decompose the variance of system evolution phylogenetic beta diversity [52]. βNTI was used as the dependent variable, with environmental and biological variables as the independent variables, and Euclidian distance was calculated. Environmental variables included soil properties. The distance matrix for the biological variables was calculated based on the associations between taxa in each soil sample.

## 3. Results

### 3.1. Soil Properties Depending on Depth

Significant vertical variations in soil properties were observed across the 0–100 cm soil profile among different grassland types (Figure S1). In AM and TG, the 0–20 cm layer showed significantly higher SWC compared with the middle layer and subsoil. Conversely, DG and GD displayed an inverse trend, with lower SWC in the upper layers. SOC and TN were markedly higher in AM and TG than in GD and DG, with no significant differences observed among the three soil layers in DG and GD (*p* > 0.05). TC and AK exhibited significant differences between the topsoil and 40–100 cm layers across all grassland types, although no notable differences were found between the middle layer and subsoil. Notably, TC was lowest in the topsoil, whereas AK reached its maximum value in this layer. BD and available AP showed minimal variation, whereas EC varied significantly within the same soil layer across different grassland types.

Critically, the interaction between grassland type and soil depth exerted a strong influence on key soil properties, including SOC, BD, TP, TN, TC, AN, EC, and AK (all *p* < 0.001; Table S1), as well as pH (*p* < 0.05). However, their interaction had no differences to AP (*p* > 0.05; Table S2).

### 3.2. Dissimilarity of Bacterial Community Diversity Depending on Depth

**Figure 2:**
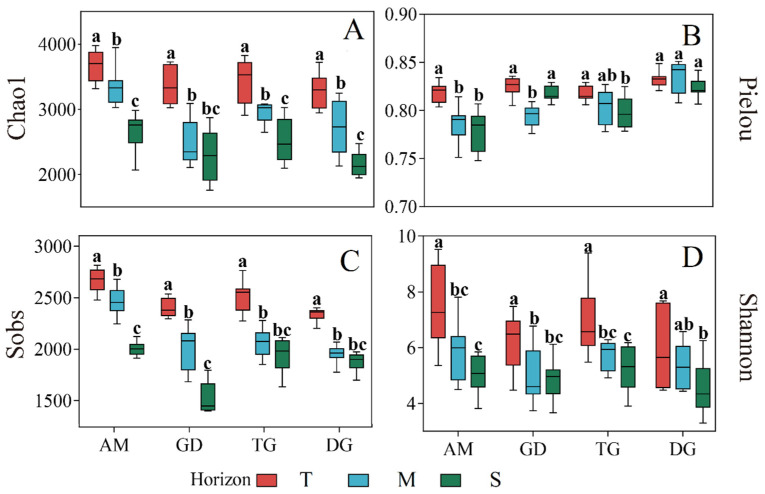
Box plot of Alpha diversity index for bacterial community in different grasslands; Chao1 (**A**); Pielou (**B**), Sobs (**C**) and Shannon (**D**). AM, alpine meadow; GD, grasslandization desert; TG, typical grassland; DG, desert grassland. Different lowercase letters indicate significant differences among different layers at the 0.05 level. T: topsoil (0–20 cm); M: middle layer (20–40 cm); S: subsoil (40–100 cm).

The bacterial community exhibited significantly higher alpha diversity in the topsoil than in the middle and subsoil layers (Figure 2). Specifically, the Chao1 index varied significantly among the four grassland ecosystems across all three soil layers (*p* < 0.05; Figure 2A), except between the middle and subsoil layers in GD, where there was no difference. Only the topsoil of AM and TG exhibited a significant difference compared to the subsoil (*p* < 0.05, Figure 2B). In contrast, there were no differences between the topsoil and subsoil in GD and DG. The Sobs index showed that the three soil layers in AM and GD differed significantly (*p* < 0.05, Figure 2C), while the middle layer and subsoil in TG and DG showed no notable distinction. The Shannon index exhibited a similar trend to the Chao1 index: the topsoil differed significantly from the middle layer and subsoil (*p* < 0.05, Figure 2D), but the middle layer and subsoil did not differ among the four grasslands. The coverage of each sample library (Good’s coverage) exceeded 0.99, demonstrating that the sequencing data comprehensively represented the microbial composition. Furthermore, the Shannon diversity rarefaction curve confirmed that the sequencing depth was adequate for capturing the full diversity across all samples (Figure S2).

**Figure 3:**
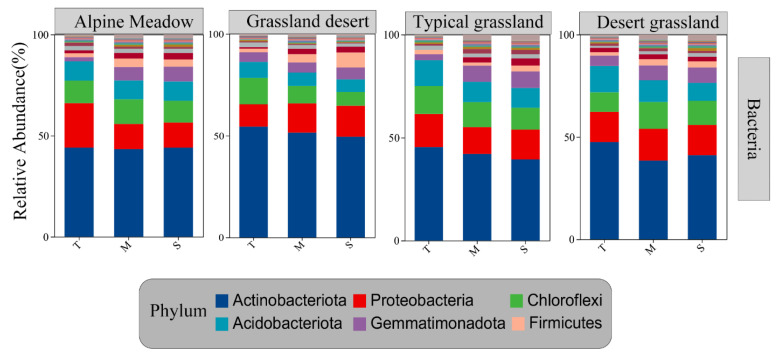
Relative abundance of bacterial community in different layers. T: topsoil (0–20 cm); M: middle layer (20–40 cm); S: subsoil (40–100 cm).

Six bacterial phyla—Actinobacteriota, Proteobacteria, Chloroflexi, Acidobacteriota, Gemmatimonadota, and Firmicutes—emerged as the dominant taxa across all soil layers (Figure 3), with their relative abundances showing remarkable horizon-dependent variations. The relative abundances of Actinobacteriota (alpine meadow: 54.4%, 51.5% to 49.5%; grassland desert: 45.4%, 42.1% to 39.4%; typical grassland: 47.5%, 41.1% to 41.0%; and desert grassland: 44.1%, 43.4% to 43.1%) decreased from the topsoil to subsoil. Meanwhile, Proteobacteria in the alpine meadow (10.9%, 14.4% to 15.2%) showed an opposite trend to grassland desert. Acidobacteriota decreased with soil depth (Figure 3).

**Figure 4:**
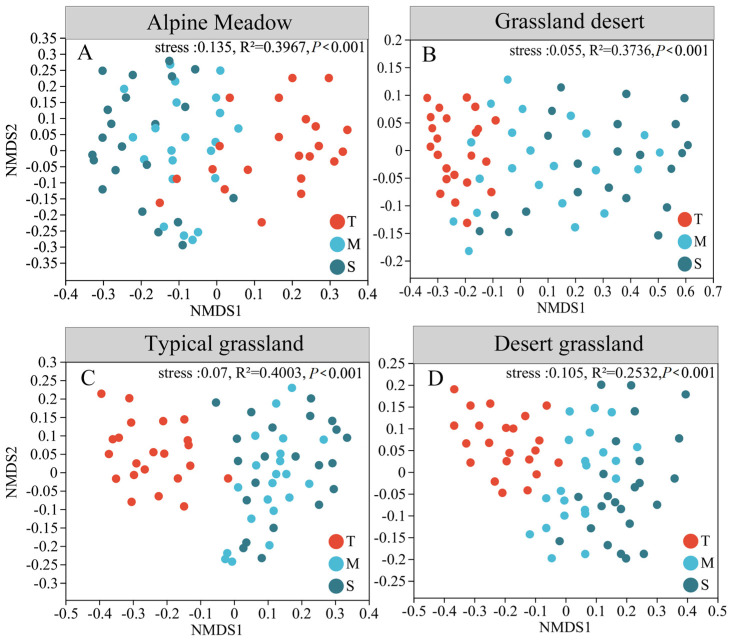
Bacterial community dissimilarity based on the Bray-Curtis distance of different layers in grassland ecosystems by NMDS analysis; Apline Meadow (**A**), Grassland desert (**B**), Typical grassland (**C**), Desert grassland (**D**). T: topsoil (0–20 cm); M: middle layer (20–40 cm); S: subsoil (40–100 cm).

The NMDS analysis based on Bray–Curtis distances revealed a distinct separation among the three soil layers (*p* < 0.05; Figure 4). In particular, the topsoil and deeper soil layers (middle layer and subsoil) showed significant difference among the four grasslands, whereas the middle layer and subsoil exhibited no difference (Figure S4). This stratification pattern was further corroborated by ANOSIM and ADONIS tests, which confirmed the statistically robust differences in bacterial communities across soil layers (*p* < 0.05; Table S3).

### 3.3. Trends in Taxon-Taxon and Taxon-Environment Networks with Soil Depth

**Figure 5:**
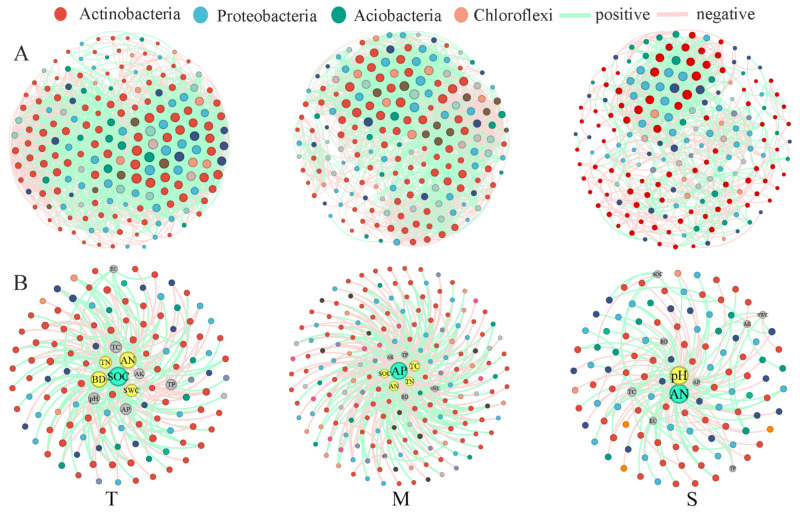
The taxon–taxon (**A**) and taxon–environment (**B**) bacterial networks across three alpine meadow soil layers are presented. In both panels (**A**,**B**), edges denote strong and statistically significant correlations (*p* < 0.01), whereas nodes correspond to unique sequences in the datasets, with node sizes scaled according to relative abundance. T (topsoil, 0–20 cm), M (middle layer, 20–40 cm), and S (subsoil, 40–100 cm).

Network analysis demonstrated that the entire soil profile, particularly the bacterial network of AM, exhibited higher connectivity, with more edges and a larger average degree compared with other grassland types (Figure 5A). Among the different grassland types, TG and GD showed intermediate network complexity, whereas DG displayed the least connected microbial associations (Figure S4). We observed a clear decline in key network metrics, including average degree, edge number, clustering coefficient, and path length, with increasing soil depth. In contrast, modularity increased with depth (Table S4). Taxon–environment association analysis revealed significant relationships between envrionmental variables and microbial communities. In AM, the bacterial community structure in the topsoil, middle layer, and subsoil was primarily influenced by three key factors: SOC, AP, and AN. In the topsoil specifically, additional contributing factors included TN, BD, SWC, and AN. In the middle layer, SOC, TC, and AN were indispensable factors, and pH also played a significant role (Figure 5B). BD, TC, pH, and AN were the crucial factors in the topsoil of GD, TG, and DG. In the middle layer, AK, TP, and SWC were the vital influencing factors for the three grasslands, whereas SOC was an indispensable factor for the subsoil (Figure S4).

### 3.4. Bacterial Community Assembly Depending on Soil Depth

**Figure 6:**
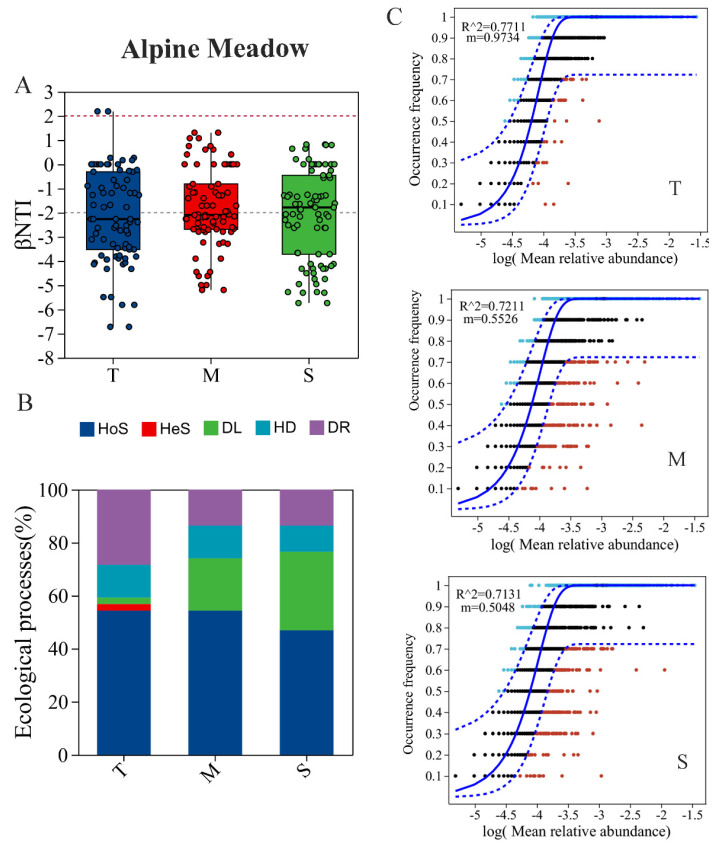
The βNTI (**A**) and the percentages of community assembly processes (**B**), and fitting of the neutral community model (**C**) of alpine meadows. The OTUs more abundant than predicted are represented by greener circles, and those less abundant are shown as red circles. The solid blue line represents the best fit for the neutral community model, with the dotted blue line indicating the 95% confidence bounds. The migration rate was estimated for “m”, and R^2^ was fitted to the neutral community model; T: topsoil (0–20 cm); M: middle layer (20–40 cm); S: subsoil (40–100 cm). HoS: homogeneous selection, HeS: heterogeneous selection, DL: dispersal limitation, HD: homogeneous dispersal, DR: drift.

**Figure 7:**
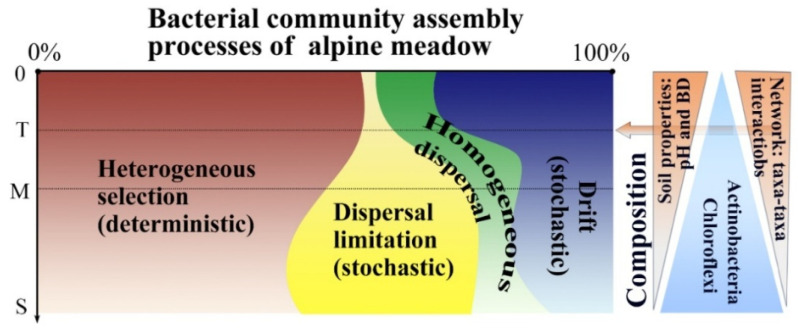
A conceptual model illustrating the bacterial community responses across different soil depths in AM. The corresponding dominance of the three main bacterial groups, and the interactions and the effects of soil properties with depth are presented on the right. The area and color shades of the graphic represent the relative importance of bacterial community assembly processes. T: topsoil (0–20 cm); M: middle layer (20–40 cm); S: subsoil (40–100 cm).

The dissimilarity in the bacterial community among sampling sites within each depth increased with geographic distance and environmental property dissimilarity (Figure S6). The results of the bacterial community analysis in AM showed that a significant proportion of the β-NTI values in both the topsoil and middle layers were below −2 (Figure 6A), indicating that deterministic processes, particularly homogeneous selection, dominated the assembly in these layers. Stochastic processes predominated in the subsoil, as evidenced by β-NTI values ranging from −2 to 2 (Figure 6B and Figure 7). In TG, the topsoil fell within the range of β-NTI < 2, indicating that deterministic processes were key. The middle layer and subsoil fell within the range of −2 < β-NTI < 2, indicating that the assembly processes were stochastic (Figure S5D,E). The assembly processes in GD were similar to those in AM across the three soil layers. In DG, the range of −2 < β-NTI < 2 in the three layers indicated that stochastic processes were the main processes (Figure S5J,K). The bacterial communities in each soil layer exhibited a good fit to the neutral model (Figure 6C and Figure S5F,I,L). The results indicate that the influence of random processes on bacterial community assembly increases with soil depth. The analysis results of the zero model are consistent with those of the neutral model analysis.

**Figure 8:**
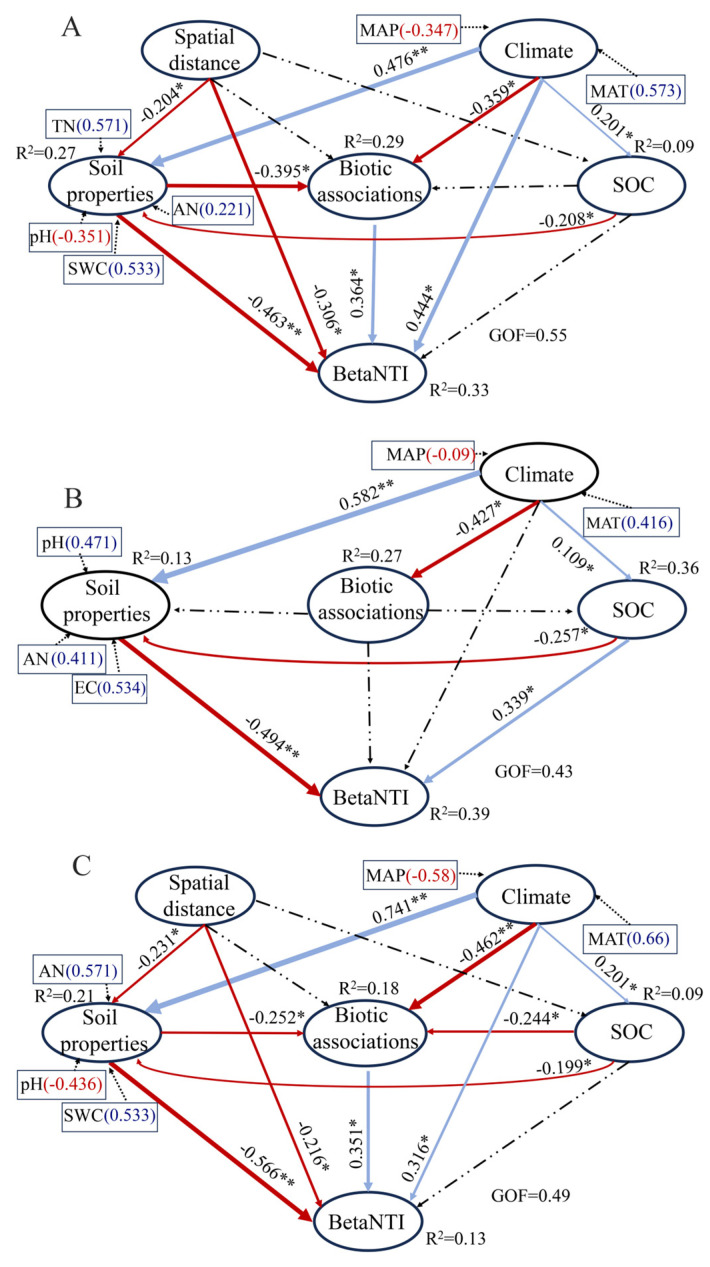
Effects of soil properties, spatial distance, biotic associations, and climates on βNTI in the topsoil (**A**), middle layer (**B**), and subsoil (**C**). The goodnesses-of-fit (GOFs) for models were 0.55 (topsoil), 0.39 (middle layer), and 0.13 (subsoil). The numbers displayed alongside arrows represent effect sizes. Positive and negative correlations are depicted by blue and red lines, whereas solid and dashed lines denote significant and nonsignificant relationships, respectively. R^2^ quantifies the explained variance. SWC: soil water content, TN: total nitrogen, AN: available nitrogen, EC: electrical conductivity, MAT: mean annual temperature, MAP: mean annual precipitation. *, ** correspond to significance levels of *p* < 0.05, and 0.01, respectively.

In the topsoil of the four grassland types, the significant factors influencing phylogenetic turnover (βNTI) were climate (MAT and MAP), soil properties (TN, pH, and SWC), spatial distance, and taxonomic associations. MAT, MAP, TN, pH, and SWC were the most important factors influencing taxonomic associations directly (Figure 8A). In the middle layer, soil properties (pH, EC, and AN) and SOC influenced βNTI. MAT and MAP influenced taxonomic associations and indirectly influenced βNTI (Figure 8B). In the subsoil of the four grassland types, climates (MAT and MAP), soil properties (AN, pH, and SWC), spatial distance, and taxonomic associations were significant factors influencing βNTI. MAT, MAP, AN, pH, SWC, and SOC were crucial factors directly influencing taxonomic associations (Figure 8C).

## 4. Discussion

### 4.1. Dissimilarity in Bacterial Community Within Soil Profiles

The most abundant bacterial phyla included Actinobacteriota, Proteobacteria, Chloroflexi, and Acidobacteriota. These phyla collectively constituted the majority of bacterial communities throughout the soil profile (Figure 4). These taxa demonstrate exceptional morphological and metabolic diversity that facilitates their widespread distribution across diverse habitats [53], a finding further corroborated by prior studies in grasslands, croplands, and forests [54,55,56]. Notably, these phyla display distinct depth-dependent distribution patterns reflecting their ecological strategies. The copiotrophic Actinobacteriota, which thrive in nutrient-rich aerobic conditions [57], decreased in relative abundance with increasing soil depth as nutrient availability decreased (Figure 4), particularly correlating with reductions in AN and AK. This contrasts with alkaline Tibetan soils, where Actinobacteria dominate in subsoils [4], underscoring pH as a master regulator of microbial distributions. Given that Acidobacteriota were more sensitive to soil acidity, and pH values increased with soil depth across the four grassland types (Figure S1), the relative abundance was higher in the topsoil, explaining their preferential abundance in topsoil. Proteobacteria are capable of utilizing SOC and nitrogen sources efficiently due to their high metabolic versatility [58]. And Proteobacteria use various substrates and can survive within a broad range of environments, from acidic and resource-limited conditions to alkaline conditions [57], thus maintaining dominance across pH and nutrient gradients. Although our study did not directly quantify microbial functional traits, the correlations between community composition and soil properties (e.g., SOC and TN) suggest depth-dependent variations in nutrient cycling potential.

This study supports our first hypothesis, demonstrating that microbial abundance, diversity, richness, and community composition exhibit significant stratification along the soil vertical profile. The diversity of the bacterial community decreased with increasing soil depth (Figure 2), which may have been caused by the decreased SOC, TN, and TP contents; this could be explained by the “resource availability hypothesis” [59,60]. And this pattern was consistently observed across diverse ecosystems, having been documented in various biomes, from temperate forests to high-elevation deserts [12,61,62]. The observed α-diversity trend shows a strong correlation with depth-dependent shifts in soil physicochemical properties, exhibiting a particularly sharp decline in the middle soil layer—a phenomenon previously documented in temperate forests and high-elevation deserts [63,64]. This consistency underscores a universal ecological principle: soil microbial diversity follows a predictable, depth-dependent attenuation in both forest and grassland systems. Crucially, the availability of resources acts as a key ecological filter [65], where variations in available nutrients and enzyme activities with depth create distinct microenvironments, posing challenges for some bacterial taxa to survive in deeper soil [31,66], this mechanistic insight explains the accelerated decline in α-diversity with depth. Furthermore, the spatial heterogeneity (β-diversity) of microbial communities is also strongly influenced by vertical gradients in soil properties [67], with significant compositional differences observed across the three soil layers (Table S3). These consistent patterns provide valuable insights for understanding soil stratification. Strikingly, this stratification pattern extends beyond grassland systems to agricultural ecosystems [68,69]. All these results indicated that differences in microbial communities in the soil layer depend largely on changes in soil environmental factors, and the soil profile exhibits a strong environmental gradient with soil characteristics changing with soil depth (Figure S1). The observed correlations between these soil parameters and the microbiome indicate that all factors exert direct or indirect effects on microbial community structure. The pronounced decrease in microbial OTU richness with depth, accompanied by consistent reductions in TN, TP, and SOC content along the soil profile, further demonstrates this pattern. Collectively, these findings evidence that vertical variations in microbial communities are predominantly driven by soil environmental factors, reinforcing the critical role of soil depth as a key determinant of microbial ecology.

### 4.2. Changes in Taxa–Taxa and Taxa–Environment Associations with Soil Depth

Co-occurrence networks provide broader insights into the mechanisms underlying species diversity across environmental gradients [70,71,72]. Studies of microbial ecological networks along biogeographical gradients have revealed significant differences in their structure across different environments [73], highlighting the importance of soil environmental context in shaping microbial interactions [74,75]. These findings also indicate the microbial community convergence in deeper soils [59], which aligns with the result of environmental heterogeneity. We further observed that the bacterial community in the subsoil was more stable, possibly due to the shared ecological niches or frequent interactions among bacteria [76]. The lower modularity in AM and GD may be attributed to limited moisture conditions throughout the soil profile, which likely inhibits microbial niche differentiation, whereas higher modularity in microbial ecology evidences more pronounced niche differentiation [77]. Several underlying mechanisms may explain the observed decline in network complexity with soil depth: the topsoil harbors a greater abundance of plant roots and root exudates, which release substantial organic carbon, thereby modulating the soil microenvironment and fostering stronger microbial interactions [78,79]. Additionally, as soil nutrients decrease with soil depth, taxa with similar physiological ecotypes tend to co-occur, leading to reduced functional redundancy [30]. And oxygen depletion in deeper soil layers may constrain metabolic energy available for niche specialization, further limiting the formation of intricate microbial network linkages [80]. And the bacterial network in the topsoil shows more positive correlations, suggesting greater ecological niche overlap and stronger positive correlations among taxonomic groups [32]. Therefore, the positive interactions and ecological niche overlap in these communities may render them more vulnerable to environmental disturbances [81]. In summary, bacterial community interactions are strongly influenced by soil depth [82].

Additionally, soil properties varied according to vegetation types, with the contents of AN and AP exerting notable impacts bacterial community structure [83,84]. Environmental variability and soil depth both exerted strong influences on microbial community composition [85]. Most bacteria were limited to specific niches by different environmental forces [86]. In this study, pH emerged as an indispensable factor in the topsoil and subsoil, significantly influencing bacterial communities across different grassland types (Figure 5 and Figure S3). pH influenced bacterial community structure by altering nutrient availability or SOC characteristics [87]. As the primary substrate for microbial activity, SOC served as a key determinant of bacterial community distribution patterns in the subsoil of AM and GD. This finding corroborates those of previous studies demonstrating SOC as a fundamental driver of microbial distribution patterns [88,89]. Multiple elements collectively shape community structures, such as EC, SWC, AK and SOC, reflecting the integrated effects of soil environmental factors [59,90]. Taxon–environment network analyses further revealed that EC, BD, and TC were the strongest correlates of bacterial taxa in the topsoil, whereas soil nutrients, SWC, and SOC played more prominent roles in the middle and subsoil layers (Figure 7). These findings suggest that soil texture in nutrient-rich layers indirectly structures microbial communities by regulating the availability of essential resources such as nutrients, water, and oxygen [30]. This aligns with the extensive literature highlighting soil nutrients, pH, and SOC as pivotal factors shaping microbial biogeography [91,92]. Therefore, future research should systematically quantify the interactions among soil pH, nutrient availability, organic matter content, and other physicochemical soil features. This would provide deeper insights into the complex relationships between microbial communities and soil environments.

### 4.3. Effects of Soil Depth on Bacterial Community Assembly Processes

The contributions of deterministic processes were generally higher than those of stochastic processes in soil up to the 60 cm layer [93]. The deterministic process of bacterial community assembly (heterogeneous selection) is amplified proportionally under extreme environmental conditions [94,95,96], such as low nutrient availability, oxygen depletion, and reduced soil density [97]. However, it diminishes significantly with depth, whereas stochastic processes, particularly dispersal limitation, become increasingly influential in middle soil and subsoils. The bacterial community assembly becomes more stochastic with soil depth, as deeper soil layers are isolated from the direct influence of environmental filtering factors (e.g., alternating flooding and drying), and are less affected by diurnal temperature dynamics [30,31,98]. This result underscores the pivotal role of environmental filtering in shaping microbial communities [62,99,100]. Topsoil bacteria could migrate into the subsoil via soil pores [93], but the subsoil exhibits higher insulation compared with the topsoil [31]. Secondly, subsoil experiences less disturbance from external factors and reduced physical disturbance in deeper layers, which inhibits bacterial dispersal. Finally, due to environmental heterogeneity, limited soil nutrients result in low dispersal rates [96]. Additionally, the stochastic assembly in subsoils suggests a higher risk of functional collapse under prolonged disturbances, as fewer taxa can compensate for functional losses. These factors explained the reasons why stochastic processes (dispersal limitation and drift) accounted for a larger proportion in deeper layers. All of the above impact may contribute to stochastic processes of bacterial community in deeper soil [101,102]. However, the effects of environmental factors exhibited a discrepancy with early studies depending on the depths of soil profiles in agricultural ecosystems [30,93,103]. Early studies on agricultural ecosystems [30,68], grasslands [31,104], and aquatic ecosystems [105,106] have evidenced that environmental selection processes dominantly drive the bacterial spatial distribution pattern on a large spatial scale (>1000 km) [107]. Although these studies have extensively explored ecological processes within bacterial communities, data on microbial community assembly in deeper soils remain scarce [25,108]. The results highlight the critical roles of both deterministic and stochastic mechanisms in driving the compositional divergence of soil microbial communities between topsoil and subsoil layers. Additionally, the bacterial community assembly process was validated through a neutral model analysis using R^2^ values and taxa located beyond the outer prediction dotted line. The parameter indicative of dispersal capacity exhibited higher values in the topsoil compared with the middle layer, while the lowest values occurred in the subsoil, implying weaker dispersal limitation in the topsoil than in the subsoil.

This study showed that soil environmental factors, spatial distance, and interspecies interactions can only explain 13–39% of the variance in bacterial community phylogenetic turnover (Figure 8). Early research evidenced that spatial distance and soil properties together explained 16% of the variance in phylogenetic turnover of the bacterial community within islands [101], a finding that aligns with this study. However, other studies have shown that environmental and distance effects alone cannot account for the majority (at least 61%) of the variation in microbial communities [109,110,111], which shows a slight discrepancy with this study. This discrepancy might occur because spatial distance is associated with environmental changes and diffusion constraints; it is difficult to completely disentangle them [52]. Also, the sampling scale, method of soil stratification, and environmental gradient of the soil profile may impact bacterial community assembly processes. Consistent with previous research, SEM was employed to elucidate the respective contributions of spatial distance and environmental factors in the bacterial community assembly [109,112]. In this study, the analysis of community assembly processes confirmed that the relative importance of deterministic and stochastic processes depends on the environment.

## 5. Conclusions

This study identifies the key environmental factors influencing microbial diversity, community composition, and vertical distribution in grassland ecosystems. The differences exhibited by bacterial communities in different soil layers can be explained to a large extent by environmental heterogeneity, rather than simply spatial isolation. Both network complexity and species interactions decreased significantly with soil depth, accompanied by a sharp decrease in nutrient content from the topsoil to subsoil, particularly for TP and TN. And topsoil exhibited a higher degree of modularity and stronger ecological connectivity than deeper layers. In addition, bacterial deterministic processes (homogeneous selection) dominated but decreased with soil depth, while stochastic (e.g., drift) increased. This represents the first study to investigate bacterial community networks and assemblages across soil depths covering a broad geographical range in Ningxia. The soil depth environment was identified as the dominant force in constructing vertical stratification of microbes. These findings advance our understanding of the subsurface ecology of microbes in grasslands and establish a framework for predicting how microbes will respond to environmental changes, providing actionable indicators (e.g., modularity and assembly process shifts) for ecosystem stability assessment. And they have important implications for biodiversity conservation and climate-resilient ecosystem management in different grassland types.

## Data Availability

The R code and datasets generated during this current study are available from the corresponding author upon reasonable request.

## Supplementary Materials

The following supporting information can be downloaded at: https://www.mdpi.com/article/10.3390/microorganisms13081934/s1, Figure S1: Characteristics of the variation in soil physicochemical properties of different layers among grassland ecosystems. SWC: Soil water content; BD: Bulk density; TN: Total nitrogen; TC: Total carbon; TP: Total phosphorus; AK: Available potassium; AP: Available phosphorus; AN: Available nitrogen; EC: Electrical conductance. Significant differences of the same grassland types in different soil layers *: *p* < 0.05; **: *p* < 0.01; ***: *p* < 0.001; Different lowercase letters indicate significant differences between different grassland types in same soil layer (*p* < 0.05). The bars in red, blue and green represent the soil layers 0–20 cm (T), 20–40 cm (M) and 40 –100 cm (S) respectively. AM: alpine meadow; GD: grasslandization desert; TG: typical grassland; DG: desert grassland.; Figure S2: Rarefaction curves of bacterial community; Figure S3: Boxplot showing the bacterial community dissimilarity in different soil horizons among grassland ecosystems. T: topsoil (0–20 cm); M: middle layer (20–40 cm); S: subsoil (40–100 cm). *, significance at *p* < 0.05 level; **, significance at *p* < 0.01 level; ***, significance at *p* < 0.001 level; Figure S4: Bacteria taxon-taxon networks and taxon-environment networks in the three layers of grasslandization desert (GD), typical grassland (TG), desert grassland (DG). The connection indicates a strong and significant (*p* < 0.01) correlation; the nodes represent unique sequences in the data sets; the size of each node is proportional to the relative abundance. T: topsoil (0–20 cm); M: middle layer (20–40 cm); S: subsoil (40–100 cm); Figure S5: The βNTI (D, I and J) and the percentages of community assembly processes (E, H and K), fitting of the neutral community model (F, I and L) of grasslandization desert (GD), typical grassland (TG), desert grassland (DG). The OTUs more abundant than predicted were represented by greener circles, less abundant were shown as red circles. The solid blue line represented the best fit for the neutral community model, with the dotted blue line indicating the 95 % confidence bounds. The migration rate was estimated for “m”, and R^2^ was fitted to the neutral community model; T: topsoil (0–20 cm); M: middle layer (20–40 cm); S: subsoil (40–100 cm). HoS: Homogeneous selection, HeS: Heterogeneous selection, DL: Dispersal limitation, HD: Homogeneous dispersal, DR: Drift; Figure S6: Distance-decay relationships between fungal community dissimilarity, environmental dissimilarity (A) and geographical distance (B) for all sites and the three soil layers. Solid lines indicate significant regressions at least at *p* < 0.05. T: topsoil (0–20 cm); M: middle layer (20–40 cm); S: subsoil (40–100 cm); Table S1: Grassland types, geographical location, and climate data of the 22 soil sites; Table S2: Effects of grassland types, soil depth, and their interaction (types × depth) on soil physicochemical properties; Table S3: ANOSIM and ADONIS test showing the differences of the microbial community structure between three soil horizons of grassland ecosystems; Table S4: Key topological features of bacterial interaction network among four grasslands.
