# Supplementary material for: Environmental Effects on Bacterial Community Assembly in Arid and Semi-Arid Grasslands
Source: Microorganisms. 2025 Aug 19;13(8):1934. doi: 10.3390/microorganisms13081934 (PMC12388656; doi:10.3390/microorganisms13081934)
Supplement: Supplementary file 1 [file microorganisms-13-01934-s001.zip › microorganisms-3804611-supplementary.pdf]

Environmental dominates bacterial community assembly in shaping spatial patterns among arid and semi-arid grasslands

Shenggang Chen[1,3], Yaqi Zhang[2,3,a], Jun Ma[1], Mingyue Bai[1], Yinglong chen,[4] Jianbin Guo[1]*, Lin Chen[3]**

[1] School of Soil and Water Conservation, Beijing Forestry University, Beijing 100083, China.

[2] School of Forestry and Prataculture, Ningxia University, Yinchuan, 750021, China.

[3] Key Laboratory for Restoration and Reconstruction of Degraded Ecosystem in Northwest China of Ministry of Education, Ningxia University, Yinchuan 750021, China.

[4] School of Earth and Environment (M087), The University of Western Australia, 35 Stirling Highway, Crawley, WA 6009, Australia.

[a] This author contribute equally to this work.

*Corresponding author: jianbinguo@bjfu.edu.cn (J. Guo)

** Corresponding author: chenlin198388@163.com (Lin Chen)

Table S1 Grassland types, geographical location, and climate data of the 22 soil sites

Grassland types	Longitude (E)	Latitude (N)	Elevation (m)	MAP (mm)	MAT (°C)	Domain vegetation
TG1	106°30′45.03″	36°45′6.57″	1973	380	7.0	Tansy(Tanacetum vulgare)
TG2	106°16′3.48″	36°24′23.8″	1950	391	7.7	Altai Hawkweed(Aster altaicus)
TG3	106°24′46.39″	36°12′17.30″	1859	432	7.0	Needlegrass(Stipa capillata)
TG4	106°48′14.38″	36°1′8.24″	1679	470	7.7	Sedge (Carex)
TG5	106°34′54.64″	36°13′56.39″	1743	444	7.2	
AM1	105°37′36.66″	36°27′6.49″	2653	384	4.6	Large-eared Saussurea
AM2	105°37′6.92″	36°13′58.70″	2492	420	5.5	Baikal Needlegrass
AM3	106°7′11.55″	35°54′56.04″	2246	457	5.1	White Wormwood (Stipa
AM4	106°13′46.53″	35°29′47.80″	2486	563	4.4	baicalensis)
AM5	106°14′15.35″	35°40′49.79″	2247	496	5.1	Meadow-rue (Thalictrum)
DG1	107°2′59.10″	38°4′58.89″	1474	285	7.6	Short-flowered Needlegrass (Stipa
DG2	106°59′50.93″	37°53′27.65″	1430	277	8.0	breviflora)
DG3	106°28′44.93″	37°26′28.69″	1362	273	9.0	Seablite (Suaeda glauca)
DG4	105°31′49.79″	36°44′59.79″	1807	290	8.0	Russian Thistle (Salsola collina)
DG5	105°25′36.86″	37°8′51.70″	1720	252	7.9	Bush Clover (Lespedeza)
DG6	105°1′40.06″	37°14′24.23″	1843	229	7.2	Intermediate Peashrub (Caragana
DG7	105°44′38.66″	37°23′26.63″	1410	228	9.4	intermedia)
GD1	106°28′14.83″	38°20′38.9″	1169	197	8.5	Komarov's Swallowwort
GD2	106°29′34.37″	38°6′44.67″	1228	220	9.2	(Cynanchum komarovii)
GD3	106°5′16.86″	37°37′50.45″	1323	237	9.1	White Spiny Shrub
GD4	105°57′47.86″	38°38′53.99″	1359	233	5.8	(Cynanchumkomarovii)
GD5	104°41′47.20″	37°25′58.14″	1652	192	8.0	Pearl Russian Thistle (salsola passerina)

TG: typical grassland; AM: alpine meadow; DG: desert grassland; GD: grasslandization desert; MAT: mean annual temperature; MAP: mean annual precipitation.

Table S2 Effects of grassland types, soil depth, and their interaction (types × depth) on soil physicochemical properties

Indexes	Treatment					
	Types		Depth		Types× Depth	
	F	P	F	P	F	P
pH	46.964	***	32.000	***	3.383	*
SOC (g/kg)	5024.378	***	18.188	***	24.590	***
BD (g/m^3)	50.471	***	14.282	***	14.282	***
TP (mg/kg)	658.215	***	29.914	***	13.363	***
TN (g/100g)	322.682	***	4105.057	***	242.740	***
TC (g/100g)	27.544	***	146.349	***	5.191	***
AP (mg/kg)	3.338	ns	0.157	ns	0.259	ns
AN (mg/kg)	6762.981	***	272.066	***	164.228	***
EC (μS/cm)	436.769	***	64.096	***	57.529	***
SWC (%)	43.420	***	1.731	ns	2.204	ns
AK (mg/kg)	284.513	***	277.757	***	42.266	***

SWC: Soil water content; BD: Bulk density; TN: Total nitrogen; TC: Total carbon; TP: Total phosphorus; AK: Available potassium; AP: Available phosphorus; AN: Available nitrogen; EC: Electrical conductance. Significant differences of the same grassland types in different soil layers *:$p<0.05$； **: $p<0.01$; ***: $p<0.001$.

Table S3 ANOSIM and ADONIS test showing the differences of the microbial community structure between three soil horizons of grassland ecosystems

Grassland	Bacterial			
	ANOSIM		ADONIS	
	R	p	F	p
Alpine Meadow	0.4279	**0.001**	6.244	**0.001**
Grasslandization desert	0.4377	**0.001**	6.668	**0.001**
Typical grassland	0.2869	**0.001**	4.708	**0.001**
Desert grassland	0.3518	**0.001**	4.760	**0.003**

Table S4 Key topological features of bacterial interaction network among four grasslands.

			Modularity	Edges	Positive links	Negative links	Degree	Path Length	Clustering Coefficient
Bacteria	AM	T	0.37	3024	1848	1176	31.336	3.514	0.564
		M	0.453	2279	1497	782	23.739	2.815	0.541
		S	0.61	830	497	333	8.829	2.546	0.523
	GD	T	0.283	1889	975	914	19.989	3.433	0.554
		M	0.472	1189	541	648	12.717	3.454	0.472
		S	0.622	679	424	255	7.341	4.132	0.429
	TG	T	0.515	1792	920	872	18.57	3.956	0.546
		M	0.549	1217	690	527	12.231	3.49	0.533
		S	0.587	698	401	297	7.386	2.958	0.428
	DG	T	0.542	1005	649	356	10.414	3.883	0.506
		M	0.534	970	536	434	10.157	3.749	0.461
		S	0.464	809	488	321	8.560	3.629	0.425

T: topsoil (0-20 cm); M: middle layer (20-40 cm); S: subsoil (40-100 cm).AM: alpine meadow, GD: grasslandization desert, TG: typical grassland, DG: desert grassland.

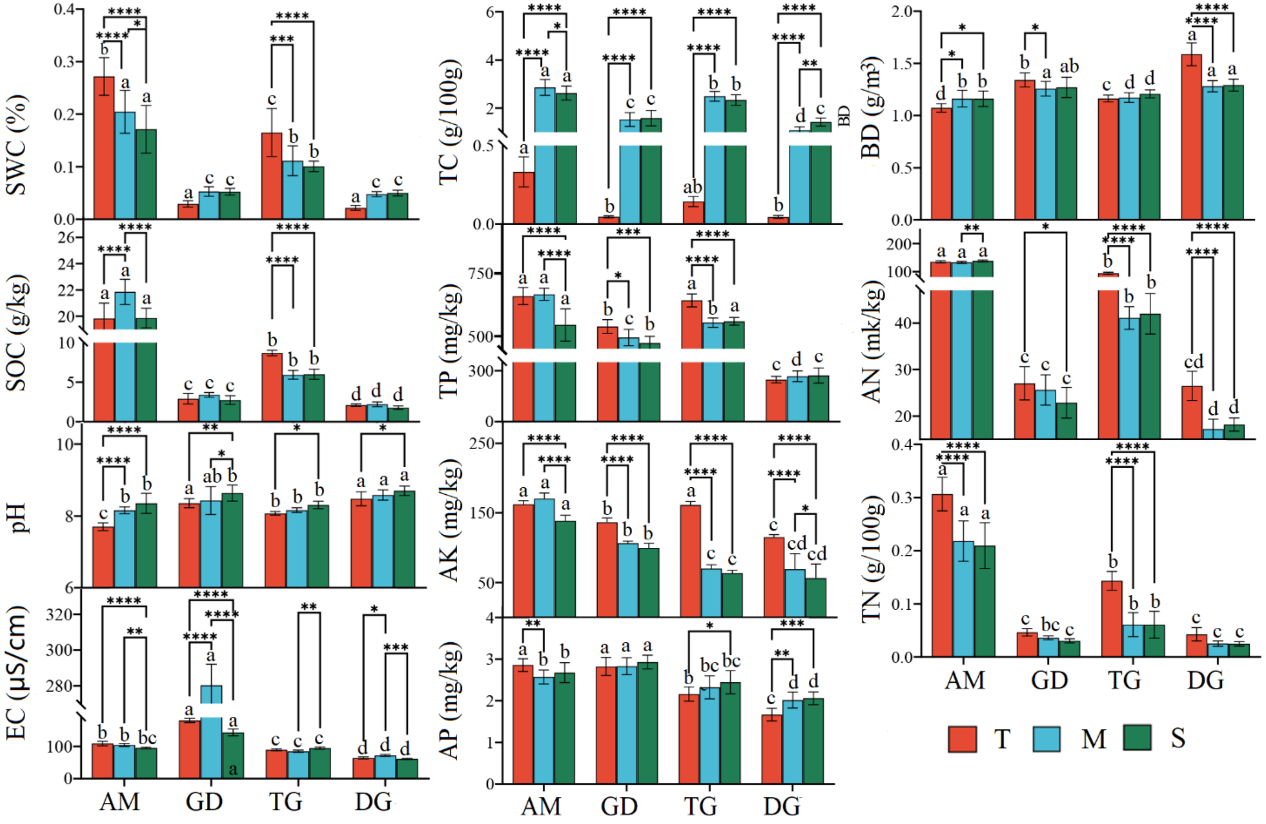

Fig. S1 Characteristics of the variation in soil physicochemical properties of different layers among grassland ecosystems. SWC: Soil water content; BD: Bulk density; TN: Total nitrogen; TC: Total carbon; TP: Total phosphorus; AK: Available potassium; AP: Available phosphorus; AN: Available nitrogen; EC: Electrical conductance. Significant differences of the same grassland types in different soil layers *:p<0.05； **: p<0.01; ***: p<0.001; Different lowercase letters indicate significant differences between different grassland types in same soil layer ($p < 0.05$). The bars in red, blue and green represent the soil layers 0–20 cm (T), 20–40 cm (M) and 40 –100 cm (S) respectively. AM: alpine meadow; GD: grasslandization desert; TG: typical grassland; DG: desert grassland.

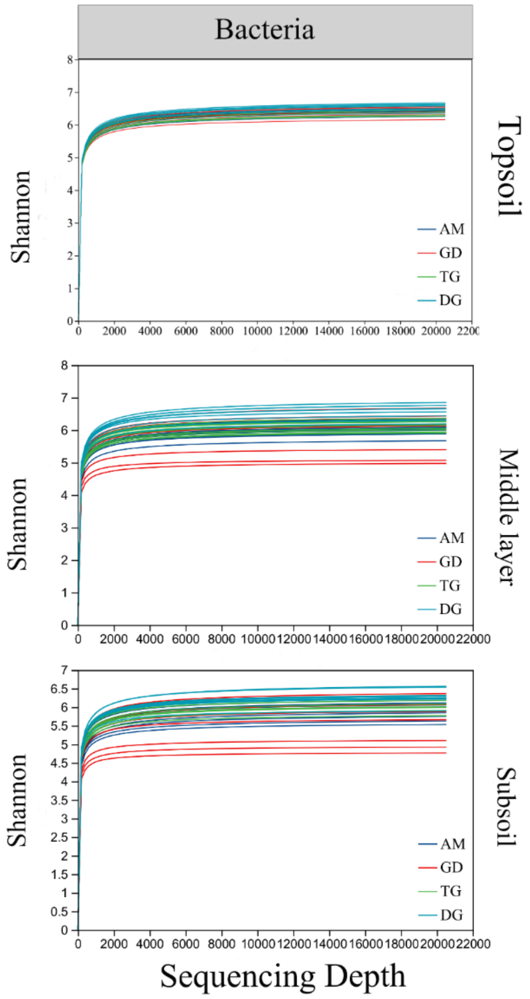

Fig. S2 Rarefaction curves of bacterial community.

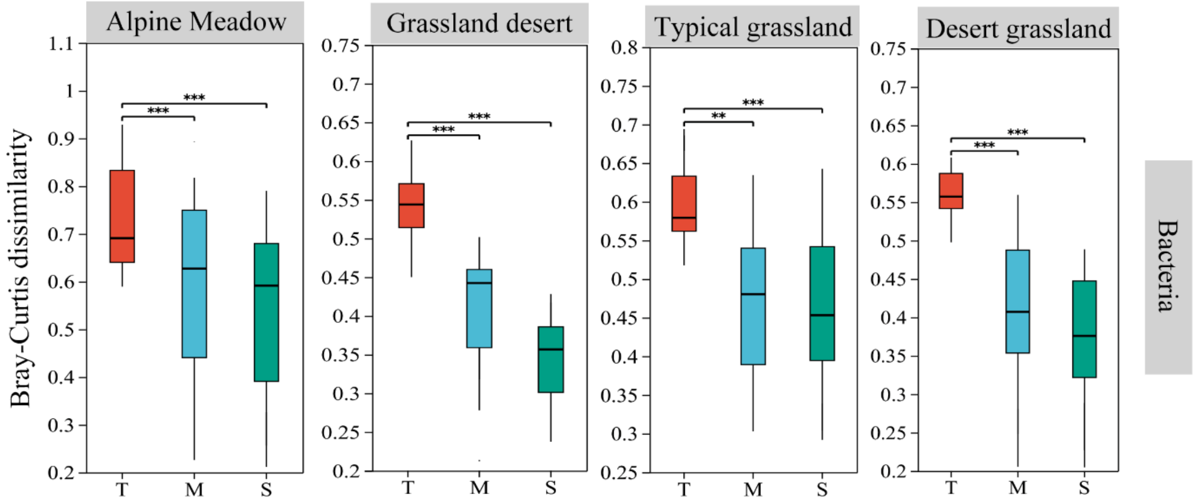

Fig. S3 Boxplot showing the bacterial community dissimilarity in different soil horizons among grassland ecosystems. T: topsoil (0-20 cm); M: middle layer (20-40 cm); S: subsoil (40-100 cm). *, significance at p<0.05 level; **, significance at p<0.01 level; ***, significance at p<0.001 level.

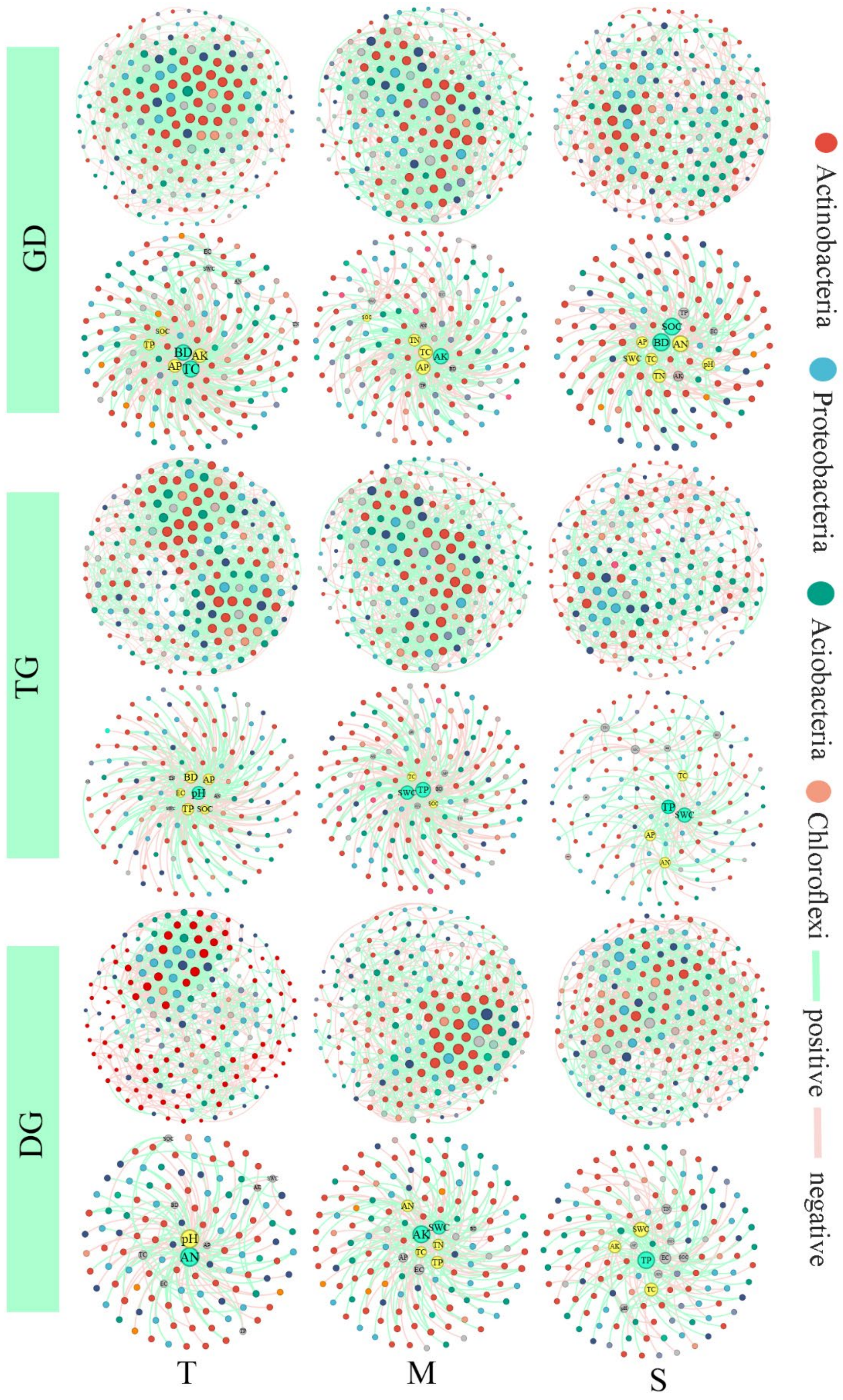

Fig. S4 Bacteria taxon-taxon networks and taxon-environment networks in the three layers of grasslandization desert (GD), typical grassland (TG), desert grassland (DG). The connection indicates a strong and significant ($p < 0.01$) correlation; the nodes represent unique sequences in the data sets; the size of each node is proportional to the relative abundance.. T: topsoil (0-20 cm); M: middle layer (20-40 cm); S: subsoil (40-100 cm).

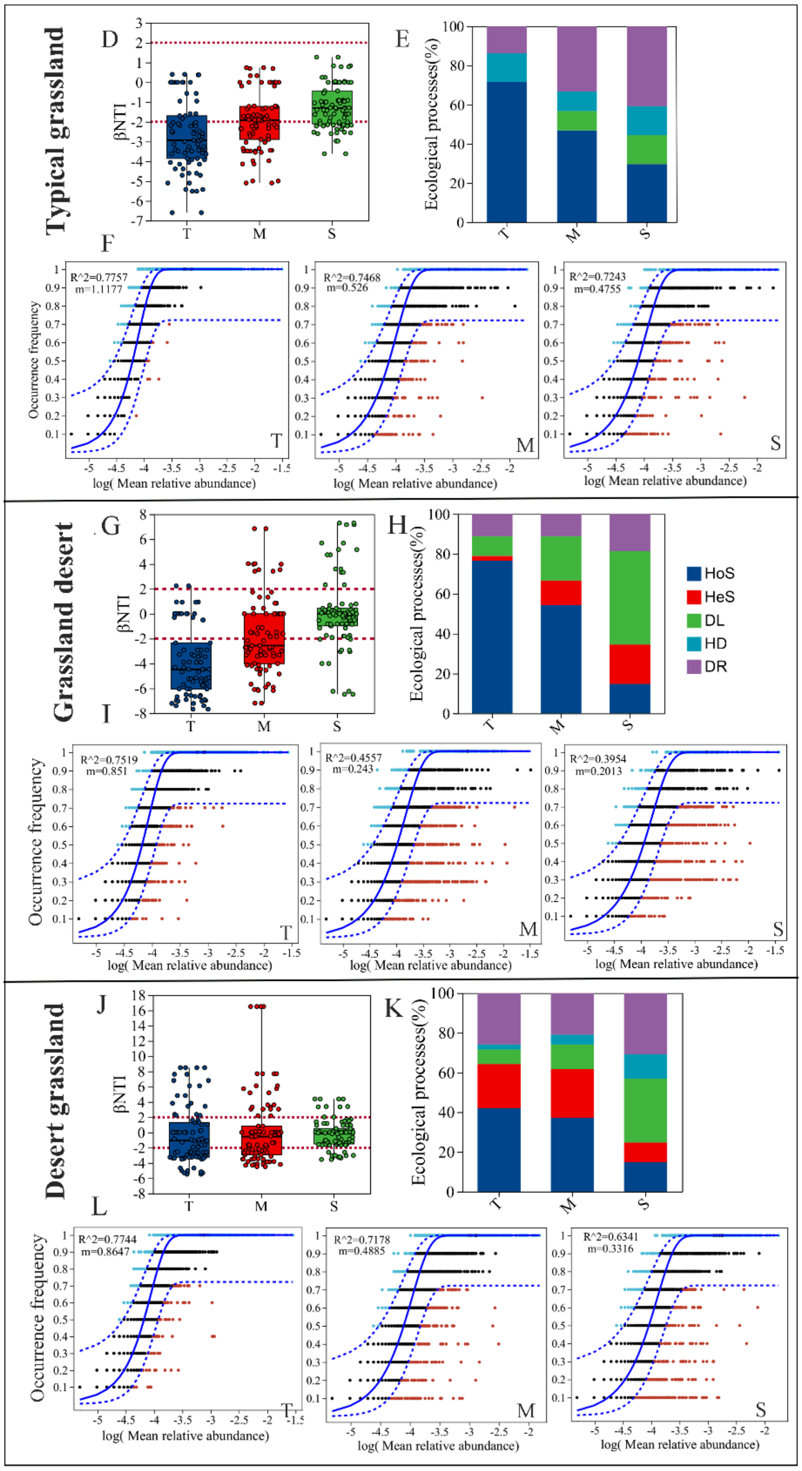

Fig. S5 The βNTI (D, I and J) and the percentages of community assembly processes (E, H and K), fitting of the neutral community model (F, I and L) of grasslandization desert (GD), typical grassland (TG), desert grassland (DG). The OTUs more abundant than predicted were represented by greener circles, less abundant were shown as red circles. The solid blue line represented the best fit for the neutral community model, with the dotted blue line indicating the 95 % confidence bounds. The migration rate was estimated for "m", and R^2 was fitted to the neutral community model; T: topsoil (0-20 cm); M: middle layer (20-40 cm); S: subsoil (40-100 cm). HoS: Homogeneous selection, HeS: Heterogeneous selection, DL: Dispersal limitation, HD: Homogeneous dispersal, DR: Drift.

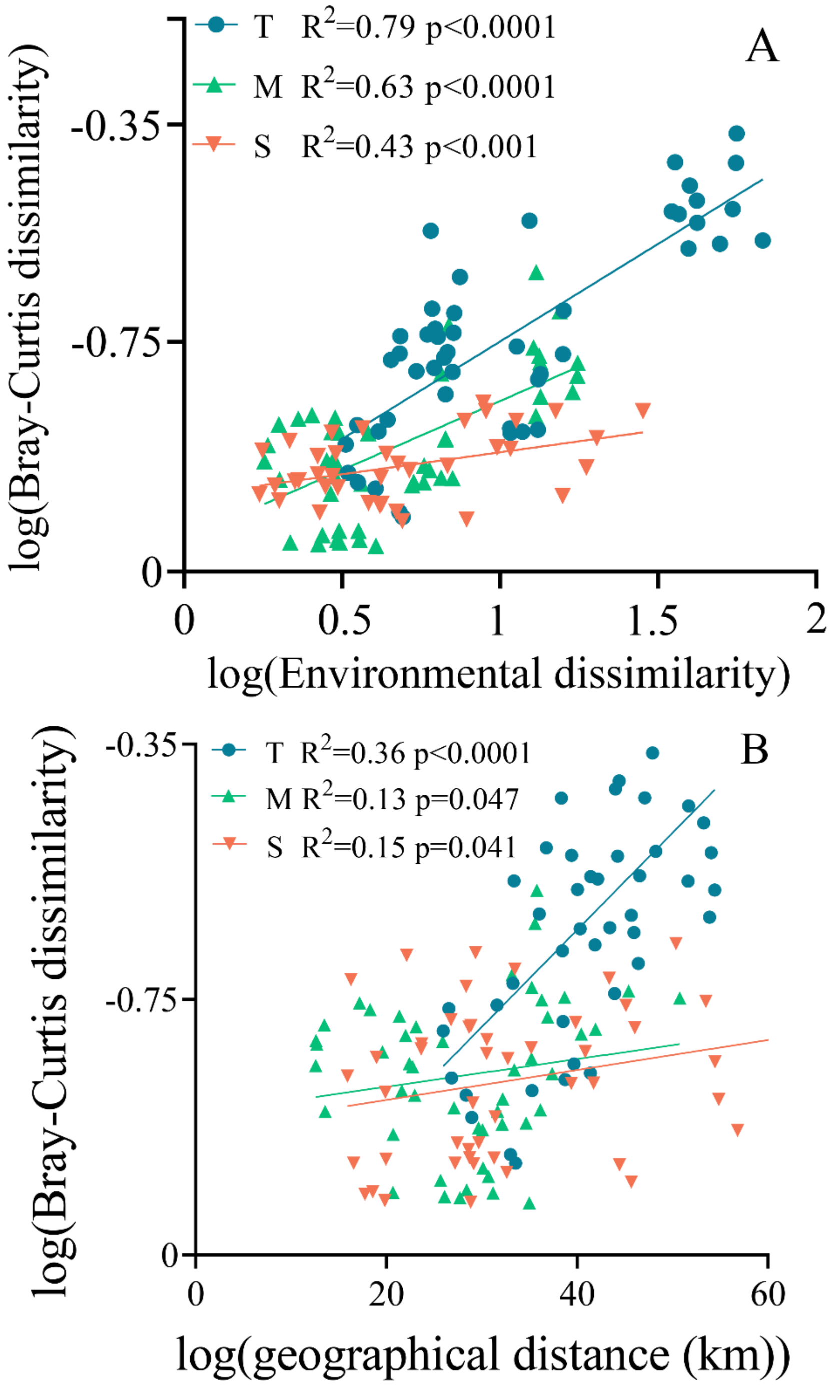

Fig. S6 Distance-decay relationships between fungal community dissimilarity, environmental dissimilarity (A) and geographical distance (B) for all sites and the three soil layers. Solid lines indicate significant regressions at least at $p < 0.05$. T: topsoil (0-20 cm); M: middle layer (20-40 cm); S: subsoil (40-100 cm).
